# Semi-Automated Air-Coupled Impact-Echo Method for Large-Scale Parkade Structure

**DOI:** 10.3390/s18041018

**Published:** 2018-03-29

**Authors:** Tyler Epp, Dagmar Svecova, Young-Jin Cha

**Affiliations:** Department of Civil Engineering, University of Manitoba, Winnipeg, MB R3T 2N2, Canada; umeppt@myumanitoba.ca (T.E.); dagmar.svecova@umanitoba.ca (D.S.)

**Keywords:** impact-echo, machine learning, artificial neural network, wavelet transformation, energy impact factor, reinforced concrete, damage detection

## Abstract

Structural Health Monitoring (SHM) has moved to data-dense systems, utilizing numerous sensor types to monitor infrastructure, such as bridges and dams, more regularly. One of the issues faced in this endeavour is the scale of the inspected structures and the time it takes to carry out testing. Installing automated systems that can provide measurements in a timely manner is one way of overcoming these obstacles. This study proposes an Artificial Neural Network (ANN) application that determines intact and damaged locations from a small training sample of impact-echo data, using air-coupled microphones from a reinforced concrete beam in lab conditions and data collected from a field experiment in a parking garage. The impact-echo testing in the field is carried out in a semi-autonomous manner to expedite the front end of the in situ damage detection testing. The use of an ANN removes the need for a user-defined cutoff value for the classification of intact and damaged locations when a least-square distance approach is used. It is postulated that this may contribute significantly to testing time reduction when monitoring large-scale civil Reinforced Concrete (RC) structures.

## 1. Introduction

Machine learning has become a larger part of SHM in recent years as the benefits of its implementation progress and knowledge of the algorithms become more widespread. Several types of machine learning exist. A non-exhaustive list of machine learning approaches includes previously mentioned Artificial Neural Networks (ANNs), Support Vector Machines (SVMs), genetic algorithms, clustering methods, Bayesian networks, and advanced deep learning [1,2]. The SVM was initially investigated as a supplement to the ANN, but was found to have a limited ability with single-feature data. The ANN was chosen because of the suitability for single-feature classification over the other methods and because of its relative ease of implementation compared to advanced deep learning.

Machine learning can be used to make binary decisions about data outputs. For the case of impact-echo data, machine learning can be used to determine damaged and intact areas based on the collected data [1,2]. The impact-echo method has been used with ANNs previously [3]. Zhang et al. (2016) used a single specimen with synthesized damages along with an Extreme Learning Machine (ELM) method to determine the location and depths of the damages [4]. The ELM differs from feed-forward neural networks by randomly connecting input and hidden neurons at the genesis of the network training [4]. The ELM was successful with limited training data in finding the location and depths of damages [4]. Sadowski et al. (2015) implemented a Principal Component Analysis (PCA) to identify pull-off adhesion between concrete layers [5]. The PCA used 80% of the collected data for training [5]. The PCA method was successful in outperforming both a feed-forward network and a multi-layer perceptron method, upon comparison of the three methods in terms of precision and flexibility. Li et al. (2014) performed automated impact-echo damage detection using a contact method on a bridge deck [6]. The results from the combination of an fast Fourier transformation (FFT) and SVM were compared to empirical results from the collected data [6]. The training of the method was accomplished using 40% of the data [6]. This produced a trained system that had a misdetection rate of 5% and a false alarm rate of 2.5% compared to the findings from the empirical results [6]. Igual et al. (2015) tested aluminum alloy specimens with various crack and hole orientations [7]. The semi-supervised method using Bayesian classification was able to identify the necessary parameters for detection with a low number of samples [7].

Stavroulakis (1998) produced numerical examples of impact-echo testing with the application of an ANN [8]. The work focused on the effects of interlaying cracks in an arbitrary elastic material. Delaminations and voids in concrete slabs were addressed in work by Xiang and Tso (2002) [9]. A contact transducer was used to collect the impact signals, and ANNs were used successfully to classify the damage types. Even with high levels of noise, the ANN correctly classified the intact, void, and delamination condition 92%, 88%, and 87% of the time, respectively [9]. Sadowski (2013) combined a Radial Basis Function (RBF) artificial neural network with the impulse-response method to find pull-off adhesion in concrete floors [10]. The RBF method was found to successfully locate the damages using the arithmetical mean height of the surface, root mean square height of the surface, surface bearing index, average mobility, and stiffness [10]. Paulraj et al. (2013) used an ANN to determine damage sites on a steel plate [11]. The study used statistical features such as kurtosis, root mean square, change in energy, and the number of slope changes in the signal [11]. The plate was excited using an impact, and accelerometers collected the signals for processing [11]. The features were then processed with an ANN and tested for the size of the training set relative to the number of test points available [11]. It was found that increasing the training set was beneficial to the performance in general, and the largest training set tested (80% of size of original data) gave the most accurate classification accuracy of 95% [11]. Zhu and Popovics (2007) located near-surface damage and the condition of ducts within a concrete slab using the impact-echo method [12]. The damages in the concrete slab imaged using the frequency response from the impacts with varying success, depending on the depth of the damage. The most successfully determined damages were 55 mm below the surface of the concrete [12]. Oh et al. (2012) showed that the impact-echo signals obtained through testing can be normalized within the signal itself; modal information can be found with the modified multipoint impact-echo (IE) testing method [13]. Sun et al. (2017) used multiple impacts to excite across fundamental frequencies and clearly visualize near surface damage [14]. Non-destructive ultrasonic waves used for damage detection are another area ANNs have been used successfully as an alternative to impact-echo testing [15,16]. Legendre et al. (2001) successfully interrogated weld locations using contact ultrasonic waves on a lab-scale specimen [15]. A 1.5-MHz transducer was used to excite the plate, and the transducer in receiver mode captured the reflected waves [15]. Wavelet transform information was used as the features for the ANN training, resulting in correct identification of weld class in 90% of the signals [15].

Autonomous testing of structures is difficult to carry out because of safety concerns. Bridge decks face unique challenges because of the vehicular and pedestrian traffic. Continuous measurement by in situ sensors such as accelerometers, strain gauges, and acoustic emission sensors has therefore been the more prevalent method of data collection for large civil infrastructure. However, the efficacy of testing via these sensors has drawbacks when looking for features such as voids, cracks, and delaminations in the structure [3]. An automated system that more readily identifies these features would further the understanding of the structure in a pseudo-real-time manner and alert engineers to the internal condition of the structure for safety and maintenance purposes.

Testing lab-developed techniques on structures in the field is an important part of concept development. Oh and Popovics (2014) tested a bridge deck using the air-coupled impact-echo method and visualized the results using a spectral-based C-scan to identify delaminations in the structure [17]. Coring was used to validate the results and showed the method was able to identify delaminations at the top surface of the reinforcing steel [17]. Hola et al. (2011) used impulse-response along with impact-echo to detect delaminations in concrete floors [18]. The impulse-response can be used up to 2000 m from the damage and is therefore an excellent tool to identify damage location which can then be precisely identified with the impact-echo method [18]. Gorzelańczyk et al. (2013) also used the impulse-response method to find the general location of damages [19]. Ultrasonic tomography was then applied to the detected damage to verify the location and determine the extent and type of the damage [19]. The method was validated on a hydroelectric dam to show the effectiveness of the method in an out-of-lab setting [19]. Hugenschmidt and Mastrangelo (2006) used Ground Penetrating Radar (GPR) on a bridge deck to determine pavement thickness, rebar spacing, and concrete cover [20]. The method was able to find pavement thickness and concrete cover with 95% and 77% accuracy, respectively [19]. Saarenketo and Scullion (2000) also looked at the use of GPR in their work. In this instance the state-of-the-art was reviewed for asphalt-paved roads analyzed using GPR [21]. The paper described frost-level, sink hole location, and washout conditions under roadways studies, among others, carried out to show the multitude of applications of the method [21]. As mentioned above, there are limited investigations carried out for automated structural damage detection using air-coupled sensors for large-scale structures. This study uses an ANN with a calculated cutoff value to find damaged regions in a reinforced concrete beam, as part of an autonomous post-processing method that removes the need for user input following the initial setup of the system. The study also employs a semi-autonomous sensing setup to ensure accurate test locations and improve testing time. Testing is carried out using an air-coupled impact-echo method on a lab-scale specimen and on a parking garage structure in the field. This study utilizes the Energy Impact Factor (EIF), outlined by Epp and Cha (2016) [22], as the single feature for the construction of the ANN.

## 2. Materials and Methods

**Figure 1:**
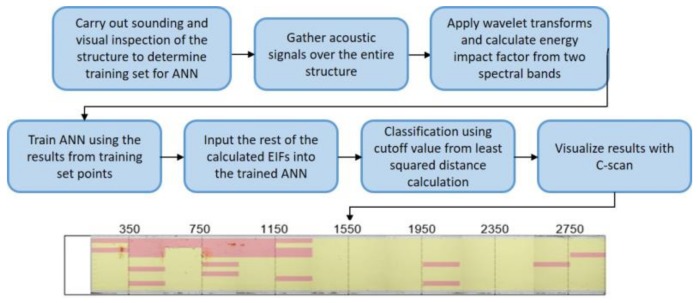
Schematic view of semi-automated damage detection.

A semi-automated sensor chassis and ANN for structural damage detection is proposed to detect internal damage of large-scale concrete structures. The schematic view of the framework is presented as shown in Figure 1. For semi-automated testing, the air-coupled impact echo method is modified to include a framework of testing devices using an Arduino circuit for automatic movement of the sensor chassis with microphones. The air-coupled impact-echo method utilizes longitudinal waves from the excitation of the structure to determine the internal state. The structure is excited using a 15.8 mm steel ball attached to a steel rod. The use of air as the coupling medium for the sensors and structure has the advantages of being easy to implement and resistant to effects from surface conditions. The drawbacks of using air as the coupling medium are that it has high impedance and limits the types of waves that can be used for investigating the structure, as not all of waves created in contact excitation readily propagate from concrete to air. Damage detection using the air-coupled impact-echo method is outlined in Figure 1.

The testing equipment used includes two picocoloumb integrated circuit piezoelectric (PCB ICP) microphones with a 20 kHz detection capability, and sensitivities of 34.4 and 41.7 mV/Pa at 250 Hz for microphone 1 (MIC 1) and microphone 2 (MIC 2), respectively. A Data Acquisition (DAQ) system was used with a sampling rate of 200 kHz on two channels to collect data from the microphones.

The use of ANNs does on some degree depend on the quality of information being used [1]. The feature used to detect damage in this study has been previously proven to provide reliable results in impact-echo testing [22]. The Energy Impact Factor (EIF) discussed in Epp and Cha (2016) is a scalar value found from the use of wavelet transformations and the ratio of high- and low-frequency coefficients from the impact-echo signal [22]. The EIF is calculated using Equation (1):

(1) $$\mathrm{Energy}\;\mathrm{impact}\;\mathrm{factor}=\frac{\mathrm{Maximum}\;\mathrm{percent}\;\mathrm{of}\;\mathrm{energy}\;\mathrm{band}\;1}{\mathrm{Maximum}\;\mathrm{percent}\;\mathrm{of}\;\mathrm{energy}\;\mathrm{band}\;2}$$

where band 1 is derived from the high frequency (4–7 kHz) information contained in the acoustic signal and decoupled using continuous wavelet transforms, and where Band 2 is derived from the low frequency (<4 kHz) information and is similarly decoupled. This work uses the Mexican Hat wavelet because of the results from previous experiments carried out by Epp and Cha (2016), but many different wavelets perform a similar function [22].

Artificial Neural Networks (ANNs) can take many forms, depending on the application [15,16,22,23,24]. In general, ANNs use features extracted from the data to develop a training network that can be used to categorize subsequent feature sets. They are particularly prevalent in the field of artificial intelligence and are becoming more prevalent in coding behind websites and apps.

Weights are initialized by scaling a random number to the range of 0–0.5 for both the input and hidden layer of the network. The back propagation and bias are also initialized and is set to 0 and 1, respectively. Once the needed parameters are set, the neural network is trained using a small subset of the collected data. For this study, a feed-forward network is used with back propagation to incorporate the gradient of the calculated error into the training [24].

The feed-forward portion of the neural network has three main components: summation of hidden layer and output layer neurons from inputs, calculation of the output of the hidden and output layers, and error calculation. The summation of hidden neurons is found with Equation (2):

(2) $$S_{j}^{\left(1\right)}=\overset{N}{\underset{i=1}{\sum }}(w_{j,1}^{\left(1\right)}x_{i}^{\left(1\right)}+w_{j,2}^{\left(1\right)}b_{i}^{\left(1\right)})$$

where $w_{j,1}^{\left(1\right)}$ and $w_{j,2}^{\left(1\right)}$ are the weight of the hidden layer neuron for the input value and bias value, respectively, $x_{i}^{\left(1\right)}$ is the input at *i*, and $b_{i}^{\left(1\right)}$ is the bias at *i*. The value of N is found from the size of the training set. The summation is carried out over all off the inputs, *i*, and applied to each hidden layer neuron, *j*. The superscript (1) distinguishes the hidden layer from output layer, which is denoted with a superscript (2).

The activation function used in neural networks can take many forms, including linear, sigmoid, and softmax functions [23]. This work uses the sigmoid activation function for the hidden and output layers because of its speed towards a classification value of one in binary classification compared with the linear and softmax functions. This work uses the sigmoid activation function for the hidden and output layers because it is computationally efficient during training step due to it being monotonic. It provides probabilistic interpretation of the output, especially for binary classification such as the damaged and intact states in this study. The softmax function is better suited for multiclass classification because of the summation of probabilities to 1. The sigmoid activation function is used to find the output of each neuron in each layer shown in general by Equation (3):

(3) $$O=1/\left(1+e^{\left(-S\right)}\right)$$

where S is the summation at each neuron for the hidden or output layer given in Equations (2) and (4), respectively.

(4) $$S_{j}^{\left(2\right)}=w_{N+1}^{\left(2\right)}b^{\left(2\right)}+\overset{N}{\underset{j=1}{\sum }}w_{j}^{\left(2\right)}O_{j}^{\left(1\right)}$$

where N is the number of hidden layer neurons and *i* is the particular output layer neuron. The summation of the output neurons is found with an updated $w_{i}^{\left(2\right)}$ and $b^{\left(2\right)}$ for each subsequent $S_{j}^{\left(2\right)}$. In other words, the $S_{j}^{\left(2\right)}$ value of j = 1 is calculated with the initial weights and bias values; the weights and bias are updated using back propagation, and the updated values are used for the j = 2 output neuron. The error, $E_{i}$, of the output neurons compared to the expected value is calculated prior to backpropagation. The calculation for the error is shown in Equation (5):

(5) $$E_{i}=\left|y_{i}-O_{i}^{\left(2\right)}\right|$$

where $y_{i}$ is the expected value of the output.

**Figure 2:**
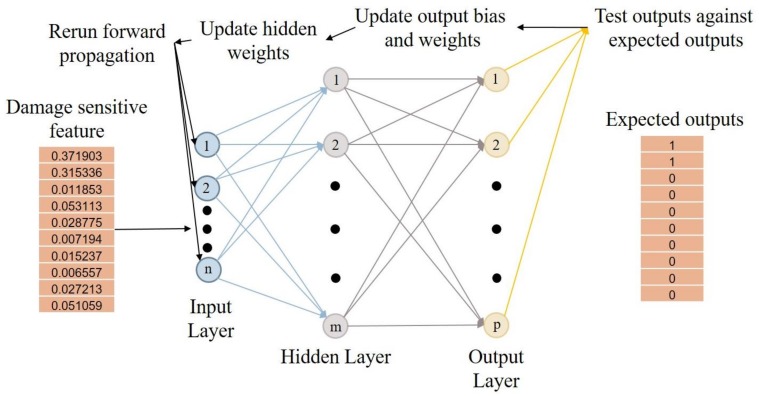
Artificial Neural Network architecture.

In both the lab and field testing, the training data are taken from the global set of EIF values describing the condition of the structure. The training sets are developed to represent intact and damaged data points. The expected values are binarized to indicate intact (0) or damaged (1) locations. Once the ANN is trained, the entire data set, including the initial training data, is run through the trained network to show the state of the structure at each test location and initiate the visualization of the results. The general layout of the ANN is shown in Figure 2.

Back propagation allows the neural network to learn from the current iteration in order to improve the direction of the subsequent iteration. It does this by updating the weights and bias values used in the feed-forward portion of the algorithm. Once the defined number of iterations is completed, the ANN is considered trained and used to classify the remaining locations on the specimen. The summation and output of the hidden layer are found using Equations (2) and (3), respectively. The output of the hidden layer is then found with Equation (4) and is used in conjunction with the sigmoid function to find the outputs correlating to each input value:

(6) $${\mathrm{Output}}_{T}=1/\left(1+e^{\left(-O_{T}^{2}\right)}\right),$$

where T is the number of test points.

The F1 score is a measure of the performance of a binary-type classification, such as the result of processing the impact-echo data. The higher the *F*1 score, the better the performance of the method is taken to be in properly identifying the expected binary values. The F1 score is a function of precision and recall of the analysis.

(7) $$\mathrm{precision}=\frac{\mathrm{true}\;\mathrm{positives}}{\mathrm{true}\;\mathrm{positives}+\mathrm{false}\;\mathrm{positives}}$$

(8) $$\mathrm{recall}=\frac{\mathrm{true}\;\mathrm{positives}}{\mathrm{true}\;\mathrm{positives}+\mathrm{false}\;\mathrm{negatives}}$$

(9) $$F1=\frac{2\times \mathrm{precision}\times \mathrm{recall}}{\mathrm{precision}+\mathrm{recall}}$$

**Figure 3:**
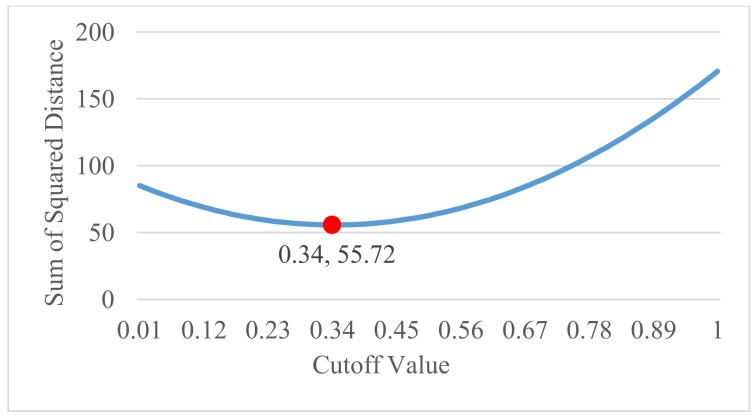
Cutoff value curve.

The cutoff value used is key to determining what information will be used to denote intact and damaged locations. The cutoff value has been automated based on the minimum squared distance from the cutoff value to the EIF values determined using the wavelet transforms. This alleviates the need for a user-defined cutoff value, but is predicated on the ability to separate the EIF values better than those first found by the wavelet transforms. The ANN fosters this by definition and filters the EIFs towards 0 (intact) and 1 (damaged) poles. Figure 3 shows the squared distance of potential Cutoff Values (CVs) of 0.01 to 1, increasing by 0.01 at every increment. Because the EIF range falls between 0 and 1, the cutoff value is tested in this range to create two groups, one damaged, and one intact. The value of the cutoff value once the ANN has been applied is not crucial because of the polarization of the data. Many cutoff values would provide the same damage detection. Automation of its selection is however valuable to remove the need for user interaction at this step in the analysis and the least-square method insures that the selection is optimal in determining the damaged and intact classes. Equation (10) gives the equation for the squared distance calculation.

(10) $$D=\sum_{k=1}^{T}{\left(x_{k}-CV\right)}^{2},$$

where $x_{k}$ is the EIF output at location k and T is the total number of EIFs.

The cutoff value is used to bin the data into intact and damaged points to produce a visual of the internal condition of the specimen. Values above the cutoff value are categorized as damaged, and values below or equal to the cutoff values are categorized as intact. The more dichotomous the data, the easier the cutoff value is to determine, because the damaged and intact points become distinct.

**Figure 4:**
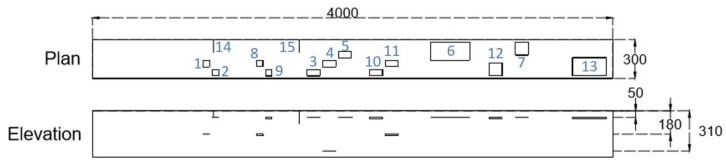
Artificially simulated damage and locations.

To validate the damage detection method, two reinforced concrete beams were tested; the first one is a simple concrete beam with artificially simulated damage with dimensions of 300 × 360 × 4000 mm, and the second one is deteriorated concrete beam in a parkade (a multistorey car park) in the city of Winnipeg. For the first beam, double-layered plastic and foam were imbedded in the concrete to simulate delaminations and voids, respectively. Delaminations and voids can provide locations for water to accumulate and cause corrosion of reinforcement. Information about delamination and void locations can therefore be important for maintenance decisions and safety of the public, if the deterioration is not addressed. The damage types and layout are seen in Figure 4 and Table 1.

The microphones were placed 2 cm from the surface of the concrete and 200 mm from the source of excitation on either side. The beam was tested at 7 rows across the short direction of the beam, with an 8th row measured only by MIC 2. Each row was tested 14 times at increments of 20 mm starting 10 mm from the edge of the beam, with the exception of the area interfered with by lifting hooks. The testing of the lab beam resulted in 126 data points from MIC 1, and 140 data points for MIC 2.

**Figure 5:**
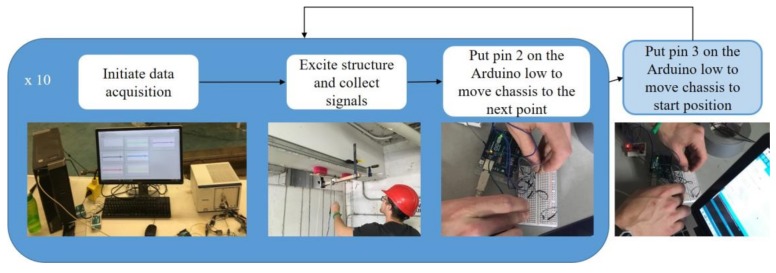
Automated test setup.

**Figure 6:**
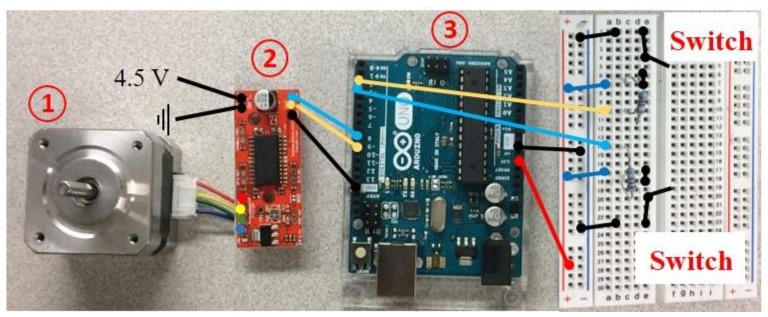
NEMA 17 motor setup.

To improve the automation, efficiency and precision of the field-test setup, a motor was installed to drive the sensor chassis as semi-automated testing device as shown in Figure 5. A national electrical manufacturers association (NEMA) 17 stepper motor, as shown in Figure 6, was used with a timing belt to enable precise movements of the impactor and microphones. The first object is the NEMA 17 stepper motor, the second is the Easy Driver, and the third is the Arduino Uno R3 board. The configuration in Figure 6 shows the interaction between the modules and is based on an EasyDriver setup [25]. Along with the Arduino R3, motor, and Easy Driver, the setup includes two pull-down resistors in series with the second and third pins of the Arduino board. The circuits with the pull-down resistors keep the second and third pins high unless the switch is closed; they connect the circuit to ground and put whichever pin the circuit is in contact with the low one. The purpose of the pull-down resistors is to limit the voltage to the Arduino Board.

**Figure 7:**
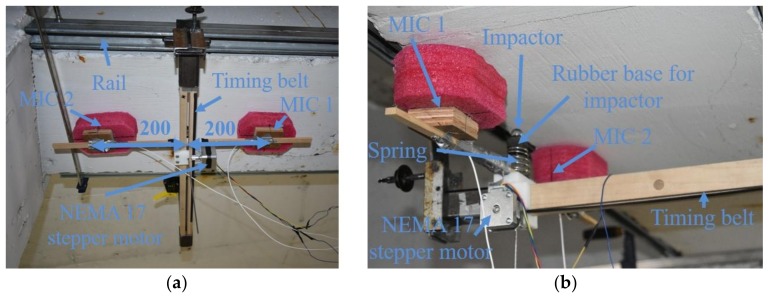
Parking garage test setup (**a**) bottom view (**b**) side view.

As a second example for a large-scale application to a real concrete structure, the experimental setup is adjusted for the orientation of the structure in the parking garage. Two rails were used to hang the sensor chassis on to avoid the need to hold the microphones and impactor overhead and to guide the chassis. A roller support was used on the opposite side of the rail to prevent vibration upon impact of the beam from the spring-mounted ball bearing. The nominal beam dimensions in the parking garage are 356 × 561 × 17,000 mm. However, testing is limited to a 2.8 m area around the visibly damaged area on the beam. The microphones were placed 2 cm from the surface of the concrete and 200 mm from the source of excitation on either side. The beam was tested at seven rows across the short direction of the beam. Each row was tested 11 times at increments of 30 mm, starting 30 mm from the edge of the beam. Foam was used to dampen the ambient noise and the isolate surface waves from the excitation as much as possible. The sensor setup is visible in Figure 7.

**Figure 8:**
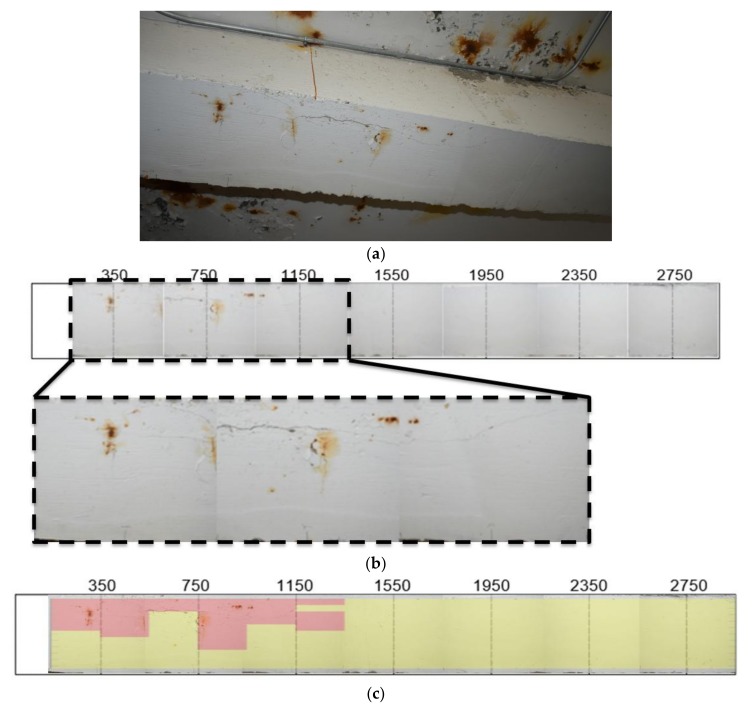
Condition of beam: (**a**) comprehensive view; (**b**) compilation of test area images for analysis; and (**c**) expected results from analysis.

The beam was visibly damaged at the time of testing. This, along with the sounding of the structure using the impactor, allowed for an approximation of damage locations to be made which could be compared to the analyzed results. The state of the beam at each of the rows is seen in Figure 8.

Figure 8b is a compilation of images taken at each of the test rows. The magnified region includes the expected damage in the upper portion of the image. The damage is generally outlined by the cracking in the concrete. The figure is used to underlie the images computed using the acoustic signals. The dashed lines in Figure 8 align with the measurements, correspond to impact sites, and otherwise correspond to microphone measurement locations. Impacts and measurements are carried out every 30 mm in the short dimension of the beam, with a spacing of 200 mm from the impact for both MIC 1 and MIC 2. Figure 7c is the expected result based on preliminary sounding and visual inspection, with the red representing the damaged region and the yellow the intact region.

## 3. Results

A single-layer ANN is subsequently applied to the air-coupled impact-echo data. Several parameters were observed to determine the most effective algorithm. Additionally, the Iteration Weight (IW), the Learning Rate (LR), the number of features, the number of neural nets, and the number of output neurons are kept constant. The number of features was kept constant because when increasing, it was found to decrease the efficacy of the ANN; the remaining features were kept constant to reduce the numbers of parameters that needed to be iterated. The number of features was determined from the previous work by Epp and Cha 2016 [22]. From the study, it was found that the EIF feature is highly sensitive to the damage. The minimal number of damage features is also beneficial to the computational cost. The number of outputs was set to one, because the problem addressed was binary classification. The iteration weight and learning rate are the parameters which should be defined by the user’s trials and errors or experience.

The acoustic signals are processed using MATLAB^®^ (Mathworks, Natick, MA, USA). The EIF discussed in Epp and Cha (2016) was used to determine the location of damage in the structure [22]. The EIF is calculated from the maximum percentage of energy from each of the two frequency bands, after signals are processed via wavelet transform. The ratio of the two maximums is then calculated and compared across the structure. A cutoff value is used to explicitly define damaged and intact regions.

The signals collected from the impact-echo testing are post-processed using the Mexican Hat wavelet transform, giving wavelet coefficients for wavelet scales 7–300. The EIF values are then calculated and used as the inputs for the ANN. The squared distance of the ANN outputs is calculated at 100 discrete values to determine the cutoff value to be used. The F1 score is then determined to compare the results from the use of an ANN and calculated cutoff value to those found from only the use of the wavelet transform. Table 2 outlines the fixed values throughout the various implementations of the ANN and Table 3 details the results from several ANN architectures that are implemented to find an optimal form from the lab experiment. The iteration weight and learning rate modulate the backpropagation updates of the ANN.

**Figure 9:**
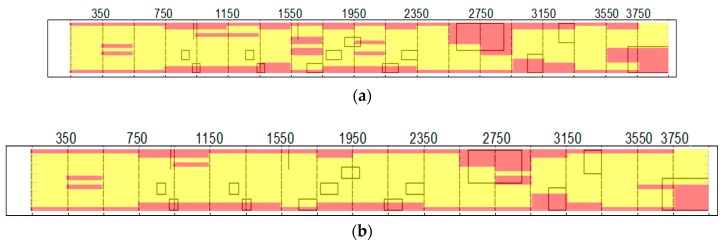
(**a**) Damage detection with 10 training points, 40 hidden layer neurons, and 1000 iterations and (**b**) automated cutoff value only.

Figure 9a shows the results of damage detection with the use of the ANN in conjunction with the cutoff value calculation. Figure 9b shows the results with the use of the cutoff value calculation only. The damaged locations are indicated in red, and the intact sections are indicated in yellow.

The F1 score of Figure 9a is 0.5829 and Figure 9b is 0.5065, working out to a performance increase of 13.1% for the neural network approach over the direct EIF data processed with a cutoff value similarly found from the least squared distance of the data. The use of a sparse training set of only 10 data points shows excellent efficiency of the algorithm and decreases the front-end work that must be done to initialize the ANN for an SHM system. The approach is predicated on a supervised approach that has damaged and intact data in the training set. The smaller the training set can be, the smaller the number of locations that need to be interrogated to perform the analysis.

The difference between the performance of the test with 1000 and 10,000 iterations for the setup with a training data set of 10 points and 40 hidden-layer neurons is a single false positive in the 1000 iteration results. Therefore, while the increase in iterations to 10,000 provides a slightly better result, the increase in runtime is substantially greater in comparison: the 1000 iteration outcomes take 13.56 s, and the 10,000 iteration outcomes take 134.23 s, using MATLAB and a Dell Precision Tower 5810 with 8 GB of Random Access Memory.

**Figure 10:**
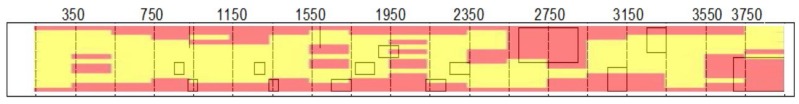
Damage detection with 34 training points, 40 hidden layer neurons, and 1000 iterations.

A larger training set of 34 points is studied in part to analyze the effect of false positives and negatives on the ANN function. Figure 10 shows the image created from the ANN run with 34 training points, 40 hidden neurons, and 1000 iterations. Of the 34 test points, 12 of the expected intact points had a higher EIF value than the lowest expected damaged point. The ANN trained was found to have a higher cutoff value and by extension more damage locations on the visual of the specimen. The F1 score of 0.5644 is reasonable and shows robustness of the ANN when used with different training sets.

The results from the parking garage testing are also analyzed using an ANN with the same architecture, as discussed previously. The constant values identified in Table 2 are held for the subsequent analysis. The cutoff value calculation used the squared distance described in Equation (10). Varying parametric values are used over multiple iterations of the analysis. Table 4 outlines the trials observed and the maximum F1 scores from each trial.

**Figure 11:**
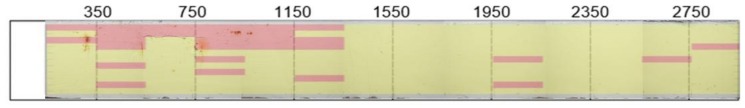
Damage detection with 10 training points, 20 hidden layer neurons, and 100 iterations.

**Figure 12:**
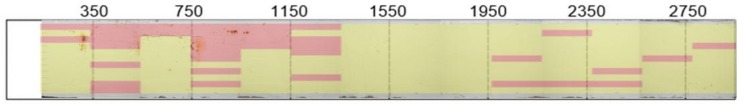
Damage detection with only EIF and automated cutoff value.

Figure 11 and Figure 12 show the results of damage detection with the use of the ANN in conjunction with the cutoff value calculation, and only the use of the cutoff value calculation on the EIF values, respectively.

The ten trials in Table 4 are consistent within the set and produce the same maximum F1 score found when a user-defined cutoff of 0.12 was used. The use of the ANN improves the F1 score by 4.1% over the results from the damage detection using only the squared distance cutoff value. This value is lower than improvement of the ANN over the squared distance in the lab specimen and can possibly be attributed to the pronounced nature of the delaminated region in the parking garage beam. Therefore, the acoustic signals gathered would be expected to be much more dichotomous in nature in the parking garage, as is the case.

**Figure 13:**
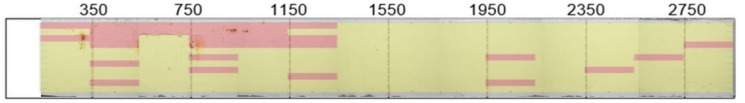
Damage detection with 34 training points, 40 hidden layer neurons, and 100 iterations.

The 20 and 36 data point training sets contain two and five false negatives, respectively. Both training sets contain three false positives. The data sets were created using sequential test points starting in the top left corner of the damage detection images. Therefore, the 36-data point set has points from the first four columns of Figure 10, Figure 11 and Figure 12. This was done to ensure expected intact and damaged points were included in the training sets. The results from the 36-data point training set, shown in Figure 13, shows the robustness of the ANN to false positives and negatives. A total of eight of the 36 data points used in training are erroneous, based on the user-defined cutoff of 0.11 that yields the maximum result, but the results differ from the maximum of 0.7368 by only 3.3%.

Within the ANN trials for the parking garage beam, certain setups showed better stability in results than others. For example, the 500-iteration and 1000-iteration trial of the architecture, with 10 inputs and 20 neurons, consistently produced results correlating to an F1 score of 0.7368. The trial with 100 iterations, on the other hand, fluctuated between F1 scores of 0.3432 and the maximum value of 0.7368. It is possible that by adjusting the learning rate or iteration weight of the algorithm, the maximum F1 score could be found more regularly. It is important to note that the result never exceeds 0.7368, but as it does reach this value, the maximum value may be used for comparison. Because of the poor results from the lab specimen algorithm which was using 100 hidden neurons, the testing for the field specimen was limited to 20 and 40 neurons.

## 4. Discussion

An air-coupled impact-echo method was proposed to find delaminations in reinforced concrete structures. The method was applied to a lab specimen and a field specimen to determine if the technique was effective in uncontrolled conditions. The testing was successful in finding near-surface damages of 100 × 100 mm and larger in the lab specimen and identified a damaged region of approximately 1200 × 120 mm in the field testing. The combination of air-coupled impact-echo testing, sounding, and visual inspection did not identify further damages in the field specimen.

The use of the ANN allows a simple distinction between damaged and intact points, which can be binarized for imaging using a simple squared distance algorithm. The trials run in this study showed that small training sets of 10 data points iterated 1000 times to update the weight and bias parameters is sufficient to create a visual of the internal condition of the beam. Additionally, when larger training set sizes were used that had at least some intact values that were larger than damaged values, the trained ANN showed reasonable robustness when the entire data set was used. The use of a training set with 34 data points iterated 1000 times with 12 values of intact locations larger than the smallest damaged location returned an F1 score of 0.5644 for the lab testing and provides the location of the damages 100 mm × 100 mm and larger. This equates to 96% of the performance of the maximum F1 score found with training sets with well-separated intact and damaged locations.

The classification value of the ANN was further shown by analyzing results from the field. A beam from a parking garage was tested using the air-coupled impact-echo method. The application of an ANN yielded the same results as those found implementing a user-defined cutoff value. Automation of the data processing for damage detection using air-coupled impact-echo testing in this way is integral in developing a system that is fully automated and driven towards creating a comprehensive testing architecture for various large civil structures. An F1 score of 0.7368 was found with a user-defined cutoff value of 0.12. Several ANN trials were able to replicate the damage identification of the user-defined setup. Moreover, sparse training sets of as few as 10 EIFs were able to train the ANN successfully. Similar to the lab testing, the field specimen training set with 36 inputs iterated 100 times had 6 values of intact locations with larger values than the smallest damaged value. The F1 score of 0.7119 found is 97% of the maximum found of 0.7368.

The use of a semi-autonomous system improved test location reliability and decreased the amount of time necessary to carry out testing. The long-term ambition of the application is the implementation of a fully autonomous system that can carry out testing and analysis without any user interaction.

As part of a long-term inspection regiment, further features, such as environmental factors, could be used to improve the performance of the method and prevent overly sensitive analysis, or more importantly, analysis that is not sensitive enough to the conditions of the structure. Future research should include testing on an in-field structure at varying temperatures, humidity, and water content to study environmental effects and to identify any need for an improvement to the robustness of the ANN. Fully automated frameworks of automation in testing should be investigated, as well as the correlation between impact on the surface condition and the efficacy of the ANN. A longitudinal study should also be considered, because it would allow data to be compared over time, and the feasibility of using an ANN with data collected from different stages of the structures’ life cycle could be determined.

## Figures and Tables

**Table 1:** Damage types and dimensions.

Damage	Type	Dimension (mm)	Depth (mm)
1	Delamination	50 × 50	180
2	Delamination	50 × 50	50
3	Delamination	100 × 50	50
4	Delamination	100 × 50	310
5	Delamination	100 × 50	50
6	Delamination	300 × 140	50
7	Delamination	100 × 100	50
8	Void	50 × 50 × 10	180
9	Void	50 × 50 × 10	50
10	Void	100 × 50 × 10	50
11	Void	100 × 50 × 10	180
12	Void	100 × 100 × 10	50
13	Void	260 × 140 × 10	50
14	Crack	100 × 50 × (<1)	
15	Crack	100 × 100 × (<1)	

**Table 2:** Fixed values for ANN computation.

IW	LR (α)	Number of Features	Number of Output Neurons
0.7	0.3	1	1

**Table 3:** Results from preliminary parameter analysis.

Training Set Size	Number of Hidden Neurons Per Layer	Iterations	F1 Score	Cutoff
10	20	100	0.5730	0.40
10	20	1000	0.5829	0.34
10	20	10,000	0.5862	0.34
10	40	100	0.5644	0.46
10	40	1000	0.5829	0.34
10	40	10,000	0.5862	0.34
20	40	100	0.5763	0.35
20	40	1000	0.5604	0.36
20	40	10,000	0.5829	0.34
20	100	100	X (all points returned damage)	-
20	100	1000	X (all points returned damage)	-
20	100	10,000	0.5683	0.37
34	40	100	0.5616	0.59
34	40	1000	0.5644	0.42
34	40	10,000	0.5614	0.34
34	100	100	X (all points returned damage)	-
34	100	1000	X (all points returned damage)	-
34	100	10,000	0.3770	0.13

**Table 4:** Results from preliminary parameter analysis from 13,000 data points.

Training Set Size	Number of Hidden Neurons Per Layer	Iterations	F1 Score	Cutoff
10	20	100	0.7368	0.11
10	20	500	0.7368	0.16
10	20	1000	0.7368	0.15
20	20	100	0.7368	0.17
20	20	500	0.7368	0.15
20	20	1000	0.7368	0.15
20	40	100	0.7119	0.16
20	40	1000	0.7368	0.15
36	40	100	0.7119	0.15
36	40	1000	0.65	0.31
